# Acute and chronic neuropsychiatric symptoms in novel coronavirus disease 2019 (COVID-19) patients: A qualitative review

**DOI:** 10.3389/fpubh.2022.772335

**Published:** 2022-08-08

**Authors:** Calen J. Smith, Perry Renshaw, Deborah Yurgelun-Todd, Chandni Sheth

**Affiliations:** ^1^Department of Psychiatry, University of Utah School of Medicine, Salt Lake City, UT, United States; ^2^Diagnostic Neuroimaging, University of Utah, Salt Lake City, UT, United States; ^3^George E. Wahlen Department of Veterans Affairs Medical Center, VA VISN 19 Mental Illness Research, Education and Clinical Center (MIRECC), Salt Lake City, UT, United States

**Keywords:** COVID-19, neuropsychiatric, depression, anxiety, PTSD, delirium, cognition, long-COVID

## Abstract

The coronavirus disease 2019 (COVID-19), caused by the severe acute respiratory syndrome coronavirus 2 (SARS-CoV-2) was declared a global pandemic by the World Health Organization (WHO) on March 11th, 2020. It has had unprecedented adverse effects on healthcare systems, economies, and societies globally. SARS-CoV-2 is not only a threat to physical health but has also been shown to have a severe impact on neuropsychiatric health. Many studies and case reports across countries have demonstrated insomnia, depressed mood, anxiety, post-traumatic stress disorder (PTSD), and cognitive change in COVID-19 patients during the acute phase of the infection, as well as in apparently recovered COVID-19 patients. The goal of this narrative review is to synthesize and summarize the emerging literature detailing the neuropsychiatric manifestations of COVID-19 with special emphasis on the long-term implications of COVID-19.

## Introduction

The novel coronavirus disease 2019 (COVID-19) caused by the severe acute respiratory syndrome coronavirus 2 (SARS-CoV-2) is spreading worldwide and the number of confirmed cases continues to rise daily posing a public health emergency. As of September 2021, a year and a half after the onset of the pandemic, there have been over 228 million confirmed cases and 4.6 million deaths globally (1). The COVID-19 syndrome primarily targets the respiratory system, resulting in symptoms such as fever, headache, dry cough, dyspnea, and dizziness (2, 3). These symptoms vary drastically from person to person, ranging from mild hypoxia to acute respiratory distress syndrome (ARDS) and sometimes death (4). SARS-CoV-2 enters human host cells primarily by binding to the cellular receptor angiotensin-converting enzyme 2 (ACE2) and by the action of the transmembrane serine protease 2 (TMPRSS2) for S protein priming (5). As treatment and mitigation efforts have progressed, there is increasing evidence that COVID-19 may have long-term system-wide health effects not limited to the respiratory system but extending to multiple organ systems including cardiac, gastrointestinal, renal, hematological, and central nervous systems, all of which express ACE-2 receptors (6–8). As an increasing number of patients have been diagnosed with COVID-19 more and more studies have shown central nervous system (CNS) involvement in COVID-19, with several studies and case reports/series demonstrating changes in mood and presence of psychological symptoms in COVID-19 patients (9). Overall, these studies suggest that neuropsychiatric symptoms may be observed in COVID-19 patients long after the infection is no longer detectable raising concerns about the long-term neuropsychiatric consequences in remitted COVID-19 patients.

There is a documented, but under-researched history of respiratory viral diseases associated with both acute and long-lasting psychopathological consequences (10). Coronavirus exposure has also been implicated in neuropsychiatric diseases during and after the severe acute respiratory syndrome (SARS) and Middle East respiratory syndrome (MERS) outbreaks (11). Coronaviruses including the SARS and MERS viruses have been shown to be potentially neurotropic, neurovirulent, and neuroinvasive (12). SARS patients who recovered reported psychiatric symptoms, including post-traumatic stress disorder (PTSD), depression, panic disorder, and obsessive-compulsive disorder (OCD) at 1–50 months follow up (13–15). Confusion and delirium were the most commonly reported symptoms during the acute stage of SARS and MERS (11). Moreover, seropositivity for coronaviruses was shown to be associated with suicide and psychosis persisting 1 year after SARS (16). Additionally, both SARS and MERS coronaviruses have demonstrated neuroinvasive potential (17, 18), with supporting evidence coming from animal studies demonstrating that SARS-CoV is capable of entering the brain upon intranasal infection of mice expressing human ACE2 (19). Thus, there is evidence suggesting that neurotropic respiratory viruses can result in chronic brain pathology that manifest as cognitive decline, mood disorders, and psychotic illness, highlighting the crucial importance of carefully tracking the neuropsychiatric sequelae of COVID-19.

The psychological impact of COVID-19 is increasingly being recognized among vulnerable groups, including health care workers, individuals in quarantine, patients with chronic medical diseases and psychiatric disorders, as well as the public in general (20). To date, there has been significant evidence demonstrating the detrimental effects of extended isolation and pandemic-related stress on mental health (21, 22). Quarantine has caused increased stress and trauma across many groups including the elderly, victims of intimate partner violence, and multi-unit households, and also affect the mental health of the general public. We focus the current review on the psychiatric sequelae of SARS-CoV-2 in COVID-19 patients (active or recovered) since it has been shown that COVID-19 patients may be especially vulnerable to depression and anxiety, second only to individuals with chronic disease with regard to point prevalence of depression and anxiety (23). The scope of the review does not include how stress related to the pandemic and social isolation due to lockdown measures adversely affects the mental health of populations that have not been infected with SARS-CoV-2.

There is increasing evidence for the psychiatric sequelae of SARS-CoV-2, though to date most research on this topic is limited to case reports/series, self-report questionnaire surveys, and mental disorder surveys (see Limitations section). Studies have reported symptoms such as anxiety, depression, and mania in COVID-19 patients. In addition, altered cognitive abilities are commonly reported. Importantly, some people who had a COVID-19 infection, even those described as “mild,” continue to suffer from persisting or cyclical respiratory and cardiovascular symptoms, as well as cognitive complaints and fatigue after the acute phase of the illness (24). This post-COVID condition is not clearly defined. It is sometimes called “post-COVID syndrome,” “long COVID,” or “post-acute COVID-19 syndrome” (25). A recent whole-brain voxel-based positron emission tomography (PET) study demonstrated brain hypometabolism in long COVID patients with biologically confirmed SARS-CoV-2 infection and functional complaints of a possible central origin, 26–155 days after the initial symptoms of infection, in comparison to healthy subjects, matched for age and sex, without antecedents of SARS-CoV-2 infection (26). There is a critical need to better characterize the individual experience as well as the biological correlates of long-COVID in order to facilitate clinicians' understanding of what is needed to help COVID patients in their recovery.

The goal of this narrative literature review is to synthesize the emerging literature detailing both the acute and chronic neuropsychiatric manifestations of COVID-19. The PubMed, PubMed Central, Google Scholar, and bioRxiv databases were searched from March 2020 to September 2021 for pertinent articles using a non-systematic approach. Common search terms included: “Psychiatry and COVID-19,” “Neurobiological Implication of SARS-CoV-2,” “Biological Mental Health and COVID-19,” and “Neuropsychiatric fallout of COVID-19.” We included articles that were in English and where study participants had a current or past diagnosis of COVID-19. Furthermore, we discuss potential neurobiological mechanisms through which the novel coronavirus may impact the CNS and result in neuropsychiatric complications. Finally, we suggest several areas of investigation that may lead to a better understanding of the impacts of COVID-19 on mental health.

## Neuropsychiatric symptoms and COVID-19

### Acute neuropsychiatric symptoms of COVID-19

Manifestations such as insomnia, psychosis, cognitive impairment, and mood disorders during the acute stage of COVID-19 infection have been described in numerous reports (Table 1). In one study, 50 (35%) of 144 patients had symptoms of anxiety and 41 (28%) had symptoms of depression, although these assessments were not diagnostic (35). In another study comparing 26 patients with SARS-CoV-2 infection with patients with other forms of pneumonia and age- and sex-matched healthy controls, the authors reported that scores on both the Hamilton Depression Scale and the Hamilton Anxiety Scale were higher for the SARS-CoV-2 group than for either of the other groups. However, these scores improved significantly after the first week of their hospital stay (53). A recently published study of 58 patients with COVID-19 who had been admitted to two ICUs in France described agitation in 40 (69%) patients and confusion in 26 (65%) of 40 patients who were assessed using the Confusion Assessment Method for the ICU. At discharge, 15 (33%) of 45 patients who were assessed had a dysexecutive syndrome with symptoms such as inattention, disorientation, or poorly organized movements in response to command (36). In the same vein, a study of hospitalized COVID-19 patients in Turkey showed that 34.9% of participants had significant levels of anxiety and 42.0% had depression at or above threshold (48). A study comparing the mental status and inflammatory markers of 103 patients hospitalized with mild COVID-19 symptoms and 103 matched COVID-19 negative controls showed COVID-19 patients demonstrated higher levels of depression, anxiety, and post-traumatic stress symptoms as assessed in online surveys (54). Levels of C-reactive protein (CRP), a peripheral inflammatory indicator, correlated positively with the PHQ-9 total score of patients who presented symptoms of depression, suggesting a potential inflammatory pathway underlying these symptoms (54).

**Table 1:** Studies investigating acute neuropsychiatric sequalae.

**References**	**Country**	**Sample size**	**Sex (F/M/Other)**	**Mean or median age**	**Patient COVID-19 status**	**Assessments**	**Key findings**
Almeria et al. (27)	Spain	35	19/16	47.6	Patients discharged from hospital (assessments performed 10–35 days after discharge)	TAVEC, WMS-IV, TMT, SDMT, Stroop, Phonemic and Sematic Fluency, Boston Naming Test, HAD	Frequent manifestations of cognitive impairment in COVID-19 patients after hospital discharge. Patients with cognitive complaints were more likely to suffer from anxiety and depression.
Abdel Azime et al. (28)	Egypt	107	56/51	41.23	10 days post infection	Self-Report Survey	100% of patients had at least one neurological symptom with headache (72%) being the most common, followed by anosmia/dysgeusia (52%), myalfia (44%), fatigue (33%) and dizziness (32%).
Beach et al. (29)	USA	4	1/3	75.25	Hospitalized	Physician Report	Delirium was a common presenting symptom in all 4 cases although $\frac{3}{4}$ patients did not present with respiratory symptoms.
Bo et al. (30)	China	714	363/351	50.2	Clinically stable hospitalized inpatients	PCL-C	96.2% of participants had prevalent post-traumatic stress symptoms before discharge.
Cai et al. (31)	China	126	66/60	45.7	Non-Infectious COVID-19 patients still in quarantine	PTSD-SS, SDS, SAS	31, 22, and 38% of participants met threshold for stress response, anxiety and depression. Older participants exhibited lower mental health symptoms as compared to younger participants
Chou et al. (32)	Multiple	3,055	1,313/1,742	59.9	Hospitalized	Self-Report and Clinician Evaluation	82% of participants had neurological symptoms.
Dravid et al. (33)	India	423			Hospitalized	Medical Record Review	Common neurological reports include headache (13.9%), dysgeusia/hypogeusia (8.3%), unsteadiness during walking (1.65%).
Ferrando et al. (34)	USA	3-case series	$\frac{1}{2}$	32.3	Hospitalized	Clinician Report	These cases all presented similarly, with new and recent-onset severe anxiety, agitation, paranoia, disorganized thinking, and none of the typical COVID related respiratory or gastrointestinal symptoms or disturbances in taste and smell
Guo et al. (54)	China	103 patients 103 controls	44/59 patients 49/54 Controls	42.5 patients 41.5 Controls	Hospitalized	PHQ-9, GAD-7, PSS-10, PCL-5,	COVID-19 patients compared to controls have higher traumatic stress, anxiety, and depressive symptoms.
Helms et al. (36)	France	140	40/100	62	COVID-19 patients admitted to the ICU	CAM, Richmond Agitation Sedation scale	84.3% of participants developed delirium, and 69.3% of participants presented with agitation. Participants presented with MRI and EEG abnormalities.
Khan et al. (37)	USA	268	119/149	58.4	Hospitalized	RASS, CAM-ICU	Delirium occurred in 29.1% of patients who did not go into a coma, 27.9% of patients expressed delirium prior to coma, and 23.1% expressed delirium following coma.
Kong et al. (35)	China	144	74/70	49.98	Hospitalized	HADS, PSSS	Anxiety and depression were observed at 34.7 and 28.4%, respectively, in COVID-19 patients. Anxiety and depression scores were significantly higher in those who were older (age > 50) and with low education. Patients with lower oxygen saturation had higher anxiety score, and those getting less social support had higher depression scores.
Li et al. (38)	USA	1,685	799/886	65.2	Hospitalized	Medical Record Review	COVID-19 Patients with a psychiatric diagnosis had higher rates of mortality compared to patients without psychiatric history.
Losee and Hanson (39)	USA	1-case report					Patient with no past psychiatric history who developed psychotic symptoms in the context of acute COVID-19 delirium.
Lu et al. (40)	China	1-case report	Male	51	Hospitalized	YMRS	Patient developed mania symptoms after vital signs had recovered (~17 days post diagnosis).
McLoughlin et al. (41)	United Kingdom	71	6/51	61	Hospitalized	Clinical Interview, AMT4	42% of participants showed symptoms of delirium.
Nalleballe et al. (42)	USA	40,469	22,258/18,211	18–50: 48.7% 51–80: 41.8% >80 = 9.5%	Active COVID-19 infection	Medical Record Review. (TriNetX database)	22.5% of participants showed neuropsychiatric manifestations. 4.6% of participants manifested anxiety related disorders, 3.8% had mood disorders, and 0.2% expressed suicidal ideation.
Parra et al. (43)	Spain	16	6/10	54.1	Active COVID-19 infection	DRS-98, spanish version, Clinical notes	All patients presented with delusions, 50% had highly structured delusions, 60% had attention disturbances, 40% had auditory hallucinations and 10% had visual hallucinations.
Parker et al. (44)	USA	1-case report	Male	57	Late Infectious Period	MoCA, Physician Clinical Assessment	Patient presented with delusions, hallucinations, and disorganized thought/behaviors requiring inpatient treatment despite no previous psychiatric history prior to COVID-19 infection.
Poloni et al. (45)	Italy	57	38/19	82.8	Active COVID-19	CDR, CAM	Delirium represented the initial manifestation of COVID-19 in 36.8% of participants. Delirium was strongly associated with mortality
Ray et al. (46)	UK	52	22/30	9	Hospitalized	Medical Record Review	COVID-19 neurology group had diagnoses including but not limited to encephalitis, Guillan-Barr syndrom, demyelinating syndrome, and psychosis. COVID-19 neurology group presented with greater rates on neuroimmune disorders compared to PIMS-TS (48 vs. <1%).
Romero-Sanchez et al., (47)	Spain	841	368/473	66.4	Hospitalized	Medical Record Review	19.9% of patients presented with neuropsychiatric symptoms. Insomnia was the most common followed by anxiety, depression, and psychosis.
Sahan et al. (48)	Turkey	281	138/143	55	Hospitalized	HADS-A, HADS-D, MADRS-S	34.9% of participants had significant levels of anxiety and 42.0% had depression at or above threshold. Hospital stay length was inversely correlated with HADS-A and HADS-D scores.
Taquet et al. (49)	USA	62,354	34,564/27,525	49.3	Hospitalized	Medical Record Review (TriNetX Analytics Network)	In the period between 14 and 90 days after COVID-19 diagnosis, 5.8% COVID-19 survivors had their first recorded diagnosis of psychiatric illness compared with 2.5–3.4% of patients in the comparison cohorts. A psychiatric diagnosis in the previous year was associated with a higher incidence of COVID-19 diagnosis
Varatharaj et al. (50)	United Kingdom	153	80/73	71	Active COVID-19 infection	Electronic Health Record Review	Altered mental status was the second most common presentation, comprising encephalopathy or encephalitis and primary psychiatric diagnoses, often occurring in younger patients. 59% of COVID-19 patients with altered mental status met criteria for psychiatric diagnosis.
Wang et al. (51)	USA	15,110	8,980/6,090/30	Not reported		Electronic Health Record Review	Patients with a recent mental health diagnosis had increased rates of COVID-19 infection with depression and schizophrenia having the highest effects. Mental health diagnosis and COVID-19 cooccurrence were associated with an increase hospitalization and death rate relative to patients without mental health history.
Xie et al. (56)	China	25 patients 55 controls	12/13 (patients) 22/33 (controls)	53.1 (patients) 40.7 (controls)	Hospitalized	ICD-10, HRSD-17, HRSA, PANSS	Insomnia, aggressive behaviors, delusions, and anxiety were all present at significant rates upon admission. Patients admitted to the psychiatric hospital had shorter hospitalizations and improved outcomes compared to controls.

One of the largest studies reported examined data from medical records of 40,469 COVID-19 positive cases, mostly from the United States of America (76%), and found that 22.5% had neurological and/or psychiatric manifestations, with anxiety and related disorders being the most prevalent (4.6%) (42). A surveillance study completed in the United Kingdom (UK) showed that 39 cases in a cohort of 125 COVID-19 hospitalized patients presenting with neurological manifestations (Coronerve National Registry Study) also displayed altered mental status. This subgroup of patients was found to have encephalopathy (*n* = 16) and neuropsychiatric syndromes (*n* = 23). One symptom that is generally limited to the acute phase of symptomology is psychosis with new-onset psychosis being reported in several studies. One study found that among neuropsychiatric syndromes, new-onset psychosis was the most common (*n* = 10) followed by other related psychiatric disorders (*n* = 7) (50). In addition, in a Spanish cohort of hospitalized COVID-19 patients (*n* = 841), about 20% of the sample were reported to develop neuropsychiatric symptoms including insomnia (13%), anxiety (8%), depression (5%), and psychosis (1.3%) (47).

There have also been case reports and case series indicating the presence of manic and psychotic symptoms in COVID-19 patients with no prior psychiatric diagnosis (29, 39). For example, a Spanish case series highlighted new-onset psychosis in several infected COVID-19 patients (52) and an American case-series described three patients who developed psychoses during infection, albeit some had a prior psychiatric history (34). Parra and colleagues reported several SARS-CoV-2 infected patients that experienced new-onset psychosis during the infection, with delusion, auditory and visual hallucinations, and orientation/attention disturbances being the most common (43). Other large case series have reported high rates of first-episode psychoses in otherwise asymptomatic patients with confirmed SARS-CoV-2 infection. For instance, Iqbal et al. reported nine cases of psychoses contemporaneous to diagnosis with COVID-19 (55). Very few studies have assessed psychiatric symptoms of COVID-19 patients by using psychiatric interviews. One such study of hospitalized COVID-19 patients in China identified 11 symptoms in this patient population, including insomnia, aggressive behaviors, delusions, and hallucinations (56). However, given the small sample size (*n* = 25), and the fact that participants were patients who received psychiatric inpatient care for comorbid first-onset mental disorders, these findings may have limited generalizability.

Delirium is the most common acute neuropsychiatric syndrome (41) and may be the most prominent presenting feature of COVID-19 in older adults and those with dementia (45). Delirium is associated with poorer outcomes (41) and is especially prevalent among patients requiring intensive care (36). Taken together, it is clear that patients with active COVID-19 infection can exhibit a range of neuropsychiatric symptoms with similar findings independent of geographical boundaries. However, the underlying pathological mechanisms have not been fully established, and a considerable number of reports in the literature remain conceptual, as studies investigating the neuropsychological effects of SARS-CoV-2 are few and limited in scale.

### Chronic neuropsychiatric symptoms of COVID-19

An emerging research base is highlighting the potential long-term neuropsychological consequences of COVID-19 infection (Table 1). In a sample of recovered COVID-19 patients at 1-month follow-up after hospital treatment in Milan, a significant proportion of patients self-rated in the psychopathological range: 28% for PTSD, 31% for depression, 42% for anxiety, 20% for obsessive compulsive symptoms, and 40% for insomnia (9). Importantly, baseline systemic immune-inflammation index (SII), which reflects the immune response and systemic inflammation, is positively associated with scores of depression and anxiety at follow-up (9). Results from this study should be considered preliminary, as these data are based on self-reported questionnaires and not on clinically assessed standardized evaluation or DSM-5 diagnoses.

In another survey-based study of COVID-19 patients (*n* = 675) who were discharged from the hospital in Wuhan, China, moderate to severe anxiety was reported by 10.4%, and moderate to severe depression was found among 19% of the sample (71). In addition, 12.4% surpassed the cutoff for provisional PTSD diagnosis, which is in contrast with the finding that 96% of patients currently hospitalized for COVID-19 showed clinically significant symptoms of PTSD due to COVID-19 (30). The presence of PTSD symptoms due to COVID-19 may be substantially higher in hospitalized patients due to acute illness and may resolve considerably after discharge. Furthermore, the study by Liu et al. noted that perceived discrimination (disconnect/rejection from family and community) was strongly associated with anxiety, depression, and PTSD (71).

Consistent with the studies above, Tomasoni et al. detected a substantial proportion of clinically and virologically recovered COVID-19 patients still suffering from anxiety (29%) and depression (11%) symptoms (68). Moreover, subjects with pathological HADS-A/D scores complained of the persistence of cognitive deficits more frequently than patients with scores in the normal range. In addition, Poyraz et al. investigated psychiatric symptomatology and protracted symptoms in patients who had recovered from the acute COVID-19 infection and found that after a mean of almost 50 days following the diagnosis, 34.5% of the sample reported clinically significant PTSD, anxiety, and/or depression, with PTSD being the most common condition reported (25.4%) (64). Predictors of PTSD symptom severity were the female sex, past traumatic events, protracted symptoms, stigmatization, and a negative view on the COVID-19 pandemic (64). A study of Italians over the age of 60 who had fully recovered from COVID-19 showed the sample experienced considerably more psychological distress than the Italian and worldwide general population, as measured by the Temperament Evaluation of Memphis, Pisa, Paris, and San Diego (61). The authors also found women to be more vulnerable to psychological distress than their male counterparts post COVID-19 infection. Patients who recovered from COVID-19 and who reported psychological distress presented with more occurrences of cyclothymic and depressive affective temperaments and scored higher on the dimensions of lack of impulse control and lack of clarity using the Difficulties in Emotion Regulation Scale (61). In a holistic study of discharged COVID-19 patients that assessed the medium-term effects of SARS-CoV-2 infection on mental and cognitive health, the authors reported that recovered patients had a higher burden of self-reported mood and dysexecutive cognitive symptoms (65). Furthermore, a large retrospective study examining COVID-19 patients in the United States of America found a bidirectional relationship between COVID-19 and mental illness, where COVID-19 diagnosis was associated with an increased incidence of psychiatric diagnosis in patients with no previous reports of mental illness (49). At 90 days, the estimated probability of having been newly diagnosed with a psychiatric illness after COVID-19 diagnosis was 5.8%. Further, a psychiatric diagnosis in the previous year was associated with a higher incidence of COVID-19 diagnosis (49). In agreement, a large case-control study based on electronic health records of patients in the US found that the odds of being diagnosed with COVID-19 were higher for patients with attention deficit hyperactivity disorder, bipolar disorder, depression, and schizophrenia (51). The authors hypothesized that this finding could be attributed to the potential lack of compliance with preventive behaviors and problems with the appraisal of health information in patients with mental health disorders. Furthermore, the authors posited that life circumstances (living in crowded hospitals or residencies) and socioeconomic disadvantage (homelessness) in patients with mental illnesses could also be contributing factors to this finding.

Relative to mood symptoms, the long-term effect of COVID-19 on cognitive function has been understudied. An investigation of recovered COVID-19 patients (10–35 days after hospital discharge), presenting with headache, anosmia, dysgeusia, diarrhea, and those who required oxygen therapy had lower scores in memory, attention, and executive function subtests as compared to asymptomatic patients (27). A T score lower than 30 was observed in memory domains, attention and semantic fluency [2 (5.7%)] in working memory, mental flexibility [3 (8.6%)] and, phonetic fluency [4 (11.4%)] (27). In addition, higher scores in anxiety and depression were found in patients with cognitive complaints (27). Further, in a sample of 71 COVID-19 hospitalized patients, those who were diagnosed with delirium during their hospitalization (42%) had lower cognitive scores on a telephone screening interview 4 weeks after discharge, although the between-group comparison did not reach statistical significance (*p* = 0.06) (41). Recovered COVID-19 patients have also exhibited reduced cognitive processing in the sustained attention domain as revealed by the continuous performance test (CPT) (70). A potential correlation between serum CRP level and reaction time in CPT was also reported, though further research is needed on the relationship between inflammatory factors and cognition (70). A recent UK study demonstrated substantial neurocognitive diminishment in mostly young adults who recovered from acute uncomplicated COVID-19 (median time after infection = 85 days) when compared to age-matched controls (69). Of note was the fact that the deficits in episodic memory, difficulties in concentration, and attention were not associated with fatigue, depression, hospitalization, treatment, viremia, or acute inflammation (69). Further, neuropsychological investigations from a German case series provide evidence of marked impairment of cognition independent of delirium and outlasting the duration of acute infection with SARS-CoV-2 (59). Finally, in the longest follow-up study of COVID-19 patients thus far, Logue et al. showed that 13.6% of participants reported persistent fatigue and loss of smell or taste, and about 2.3% of the participants reported brain fog or cognitive problems for as long as 9 months after illness (72). These results suggest that recovered COVID-19 patients may present with reduced cognitive function which may have important implications for returning to the demands of school and work Table 2.

**Table 2:** Studies investigating chronic neuropsychiatric sequalae.

**Reference**	**Country**	**Sample size**	**Sex (F/M/Other)**	**Mean or median age**	**Patient COVID-19 status**	**Assessments**	**Key findings**
Buonsenso et al. (57)	Italy	129	62/67	11	Recovered	Parental report in ISARIC survey	Patients reported 10.9% more fatigue that prior to infection. 18.6 and 10.1% of patients also reported insomnia and headaches, respectively.
Gramaglia et al. (58)	Italy	238			3–4 months post discharge	MINI, BAI, IES, BDI-II	Participants presented with 32.9% and 29.5% anxiety and depression symptoms, respectively. COVID-19 severity was not correlated with acute COVID-19 severity.
Groiss et al. (59)	Germany	4-case series	0/4	59.5	Severe COVID-19 hospitalized patients	MoCA, SDMT, MMSE	All patients had sustained cognitive impairment outlasting the acute phase of the disease for weeks.
Hampshire et al. (60)	United Kingdom	84,285 (*n =* 361 for positive COVID-19 diagnosis)	46,449/37,478/358		Recovered	Great British Intelligence Test	Individuals who recovered from suspected or confirmed COVID-19 perform worse on cognitive tests in multiple domains. This deficit scales with symptom severity and is evident amongst those without hospital treatment.
Iqbal et al. (55)	Qatar	50	2/48	39.5	COVID-19 patients referred to a consultation-liaison psychiatry service	Medical Record Review	Principal psychiatric diagnoses included delirium (26%), mania (16%), depression (16%), psychosis (18%), acute stress reaction (16%), and anxiety disorder (16%).
Janiri et al. (61)	Italy	43	14/29	67.98	Recovered	DERS, TEMPS-A-39, K10	29.51% of participants reported psychological distress. Women were more likely to experience distress than men. Patients who recovered from COVID-19 and who reported psychological distress presented with more occurrences of cyclothymic and depressive affective temperaments and scored higher on the dimensions of lack of impulse control and lack of clarity.
Kingstone et al. (24)	United Kingdom	24	19/5	42.79	Recovered	Interview	Brain fog, headaches, fatigue, are reported during COVID-19 recovery.
Logue et al. (72)	USA	234	213/162	52.6	Recovered	Medical Record Review and Questionnaire	13.6% of participants reported persistent fatigue, and loss of smell or taste. 2.3% of the participants reported brain fog or cognitive problems.
Matos et al. (62)	Brazil	7	6/1	42.5	Discharged (at least 60 post discharge)	MMSE, MoCA, CDT	Neurological manifestations of COVID-19 developed ~16 days after initial symptomology. Cognitive dysfunction was present in all participants at time of testing.
Mazza et al. (9)	Italy	402	137/265	58	Recovered	IES-R, PCL-5, Zung SDS, BDI-13, STAI-Y, MOS-SS, WHIIRS, OCI	55.7% of participants scored within the clinical range for one psychopathological measure, 36.8% scores for two, 20.6% for three and 10% for four. Females, patients with a positive previous psychiatric diagnosis, and patients who were managed at home showed an increased score on most measures.
O'Keefe et al. (63)	USA	290	216/72	42	1–6 Months post discharge	Self-Report Survey	Mental fatigue (13.5%) and fatigue (20.3%) are commonly reported symptoms in the sample.
Poyraz et al. (64)	Turkey	284	139/140	39.7	Recovered	IES-R, HADS, PSQI, Mini Suicidality Scale	34.55% of participants demonstrated clinically significant anxiety, depression, and/or PTSD with PTSD being the most common (25.4%)
Raman et al. (65)	UK	58 patients 30 controls	24/34 (patients) 12/18 (controls)	55.4 (patients) 53.9 (controls)	Recovered	PHQ-9, GAD-7, Montreal Cognitive Assessment (MoCA)	At 2–3 months, patients had higher cumulative self-reported symptom scores for depression and anxiety. Cognitive performance in the executive/visuospatial domain was impaired among patients.
Sigfrid et al. (66)	UK	327	135/92	59.7	3 Months post discharge	WG Short Set, EQ5D-5L	Fatigue was extremely common, presenting in 83% of the sample, and was independent of age and comorbidities. Anxiety, depression, and pain were worsened according to participants, female sex was significant in the increase of symptoms.
Stephenson et al. (67)	UK	3,065	1,945/1120		3 Months post infection	ISARIC Survey (Including EQ-5D-Y)	Tiredness and headaches were higher in COVID-19 positive sample. Both COVID-19 positive and negative participants reported high rates of worry, sadness, and unhappiness (~40%).
Tomasoni et al. (68)	Italy	105	28/77	55	Recovered	HADS-A, HADS-D, MMSE	29% of participants experienced anxiety symptoms and 11% experienced depressive symptoms. 17% of the recovered patients presented with cognitive symptoms
Woo et al. (69)	Germany	18 patients and 10 controls	10/8 (patients) 4/6 (controls)	Controls (38.4) Patient (42.2)	Recovered	TICS-M, PHQ-9	78% of patients reported sustained mild cognitive deficits and performed worse in the TICS-M compared to 10 age-matched healthy controls.
Zhou et al. (70)	China	29 patients 29 controls	11/18 (patients) 12/17 (controls)	47 (patients) 42.5 (controls)	Recovered	TMT, SCT, CPT, DST	COVID-19 patients scored lower on the 2nd and 3rd parts of the CPT, reaction times were also lower in the 1st and 2nd part of the CPT in COVID-19 patients.

AMT4, Abbreviated Mental State 4; BAI, Beck Anxiety Inventory; BDI, Beck Depression Inventory; CAM, Confusion Assessment Method; CDT, Clock Drawing Test; CDR, Clinical Dementia Rating; CPT, Continuous Performance Test; DERS, Difficulties in Emotional Regulation Scale; DRS, Delirium Rating Scale; DST, Digital Span Test; EQ5D, European Quality of Life 5 Dimension; GAD-7, Generalized Anxiety Disorder-7; HADS-A, Hospital Anxiety Rating Scale; HADS-D, Hospital Depression Rating Scale; IES, Impact of Events Scale; ISARIC, International Severe Acute Respiratory and emerging Infection Consortium; K10, Kessler 10 Psychological Distress Scale; MADRS, Montgomery-Asberg Depression Rating Scale; MINI, Mini-international neuropsychiatric interview; MMSE, Mini Mental State Examination; MoCA, Montreal Cognitive Assessment; MOS-SS, Medical Outcomes Study Sleep Scale; PCL-C, PTSD Checklist-Civilian Version; PCL-5, PTSD Checklist for DSM-5; PHQ-9, Patient Health Questionnaire 9; PSQI, Pittsburgh Sleep Quality Index; PSSS, Perceived Social Support Scale; PTSD-SS, post-traumatic stress disorder self-rating scale; OCI, Obsessive-Compulsive Inventory; RASS, Richmond Agitation-Sedation Scale; SAS: self-rating anxiety scale; SCT, Sign Coding Test; SDS, Self-rating depression scale; SDMT, Symbol Digit Modalities Test; STAI-Y, State-Trait Anxiety Inventory form Y; TAVEC, Test de Aprendizaje Verbal Espa~na-Complutense; TEMPS A-39, Temperament Evaluation of Memphis, Pisa, Paris, and San Diego; TICS-M, Modified Telephone Interview for Cognitive Status; TMT, Trail Making Test; WHIIRS, Women's Health Initiative Insomnia Rating Scale; WMS-IV, Visual Reproduction of the Wechsler Memory Scale –IV; YMRS, Young Mania Rating Scale.

### Impact of ICU admissions in COVID-19 on mental health

As of late December 2020, roughly 326.7 COVID-19 cases out of 100,000 resulted in hospitalization in the United States of America (CDC, 2020). The relationship between hospitalization, particularly the need for ICU care and COVID-19 may signal greater compromise in patient mental and cognitive health. For instance, a study on patients admitted to the ICU (*n* = 116) showed that 41.4% of patients reported at least one long-term mental health consequence within 6 months of discharge (73). Out of the patients experiencing mental health consequences, anxiety and depression (or the combination of the two) were the most prominent syndromes, at 28.4 and 20.7% of patients, respectively (73). In a separate study that included ICU patients receiving mechanical ventilation, symptoms of anxiety and depression were evident at 3 months follow-up in ~30 and ~21%, respectively, as well as PTSD in 29.9–34.3% of patients (74). Patients in the ICU with ARDS and severe illness (91% on ventilation) had average global cognition scores 1.5 standard deviations (*SD*) below the age-adjusted mean population and appeared similar to patients with mild cognitive impairment (75). These findings suggest that if patients admitted to the ICU with COVID-19 follow these initially observed trends, they may experience the virus's effects for extended periods. Thus, examination of the relationship between ICU admission and illness severity in COVID-19 patients will be an important area of research to determine what factors may contribute to the long-term effects on cognitive and mental health.

## Potential neurobiological mechanisms

The burgeoning body of literature on COVID-19 infection suggests it is associated with acute and chronic neuropsychiatric sequelae; however, the mechanisms underlying this association are not completely understood but appear to be multifactorial. There are several proposed biological mechanisms including direct neuroinvasion, cytokine network dysregulation, post-infectious neuronal autoimmunity and acute neurovascular events as well as psychological and environmental mechanisms such as social isolation that may play a role in the emergence of these syndromes (76, 77). There are complex challenges in assessing the true role of SARS-CoV-2 in neuropsychiatric disease, the most pertinent of which is separating the specific impact of the virus from both the broader disease response and the social, cultural and psychological circumstances in which an infection has occurred (77).

Concerning direct neuroinvasion, human autopsy studies have identified viral RNA transcripts in brain tissues (78–80) and viral proteins in the endothelial cells within the olfactory bulb (81) in people who succumbed to COVID-19. There have been several reported cases of SARS-CoV-2 presence in the cerebral spinal fluid (CSF). For instance, in one case, the presence of the viral genome of SARS-CoV-2 was detected using deep sequencing in the CSF of a patient presenting with CNS demyelinating disease (82). In another case, a COVID-19 patient with meningitis had SARS-CoV-2 presence in the CSF confirmed using polymerase chain reaction (PCR) (83). Additionally, a recent study using human induced pluripotent stem cell (hiPSC) lines derived from healthy individuals to generate forebrain-specific human neural progenitor cells (hNPCs), indicated that SARS-CoV-2 can infect cells of neural origin, and suggested that infected cells can promote the death of nearby cells (84). One of the most frequently proposed routes of neuroinvasion is through the olfactory bulb. In a study of 32 COVID-19 patient autopsies, SARS-CoV-2 RNA was found in the olfactory mucosa of 13 patients, and 3 patients displayed detectable levels of SARS-CoV-2 RNA in the olfactory bulb (85). In the same study, axonal damage was noted in the olfactory area, as well as SARS-CoV-2 RNA in connected areas such as the cornea, uvula, trigeminal ganglion, and medulla oblongata, suggesting, that axonal transport originating in the olfactory bulb could be a route of neuroinvasion of SARS-CoV-2 (85). Overall, this study provided evidence that SARS-CoV-2 may enter the CNS via trespassing the neuro-mucosal interface in the olfactory mucosa by exploiting the close vicinity of olfactory mucosal and nervous tissue, including delicate olfactory and sensitive nerve endings.

**Figure 1:**
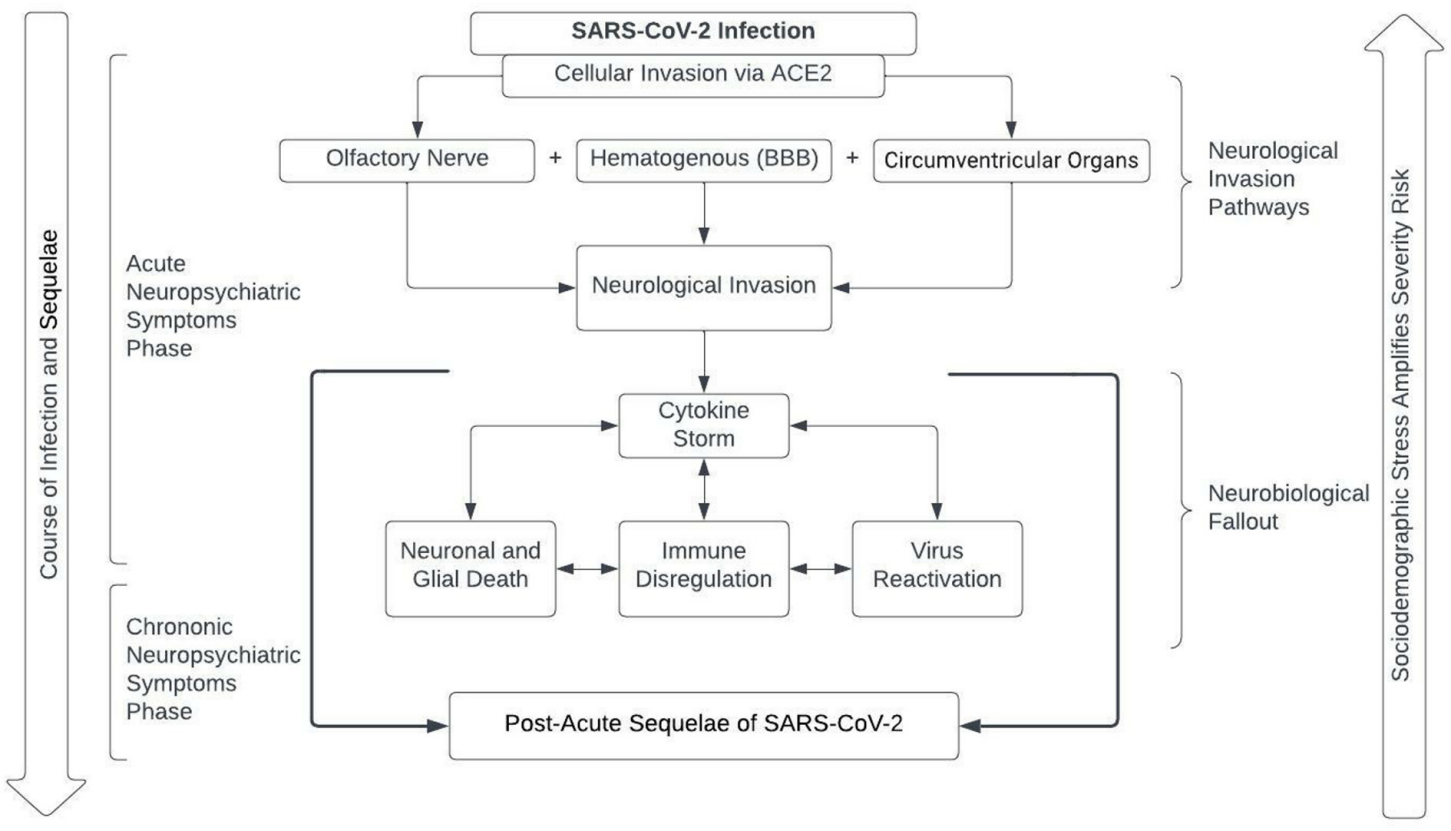
Potential etiology of COVID-19 neuropsychiatric symptoms. Following infection with SAR-CoV-2 there are several mechanisms that can potentially lead to a viral neurological invasion. The subsequent neurological invasion increases the already present cytokine storm leading to a feedback loop of biological consequences including neuronal/glial death, immune dysregulation, and virus reactivation; consequences that may potentially worsen symptomology of infection. The fallout of neurological invasion may account, at least in part, for chronic symptomology. Additionally, the risk and severity of the discussed fallout may be amplified by sociodemographic stress.

Inflammation caused by neuroinvasion and peripheral immune response to SARS-CoV-2 has the potential to induce brain changes that can be associated with increased risk for psychiatric consequences. Previous studies have shown that peripheral immune response and inflammation can cause and exacerbate acute and chronic neuroinflammatory responses (86–91). SARS-CoV-2 infection is also reported to trigger a cytokine storm whose effects on the CNS may have unpredictable consequences in both the short and the long-term. Related to the cytokine storm is the suggested reactivation of viruses such as Epstein-Barr virus, herpes simplex virus 1, and varicella-zoster virus; the reactivation of dormant viruses may prolong inflammation and, as such, prolong symptomology (92, 93). Numerous neurological and psychiatric diseases are known to have a neuroinflammatory component involving the same factors that are stimulated during the final stage of COVID-19. Of particular interest is the impact that these molecular mechanisms may have on the development and progression of neurodegenerative diseases as well as on psychiatric disorders, especially affective disorders, whose pathogenesis were found to involve neuroinflammatory mechanisms (94). Although the evidence supporting a causal role for COVID-19 associated neuroinflammation as it relates to psychiatric disease is limited, it is plausible that COVID-19 patients with a neuroinflammatory signature may be vulnerable to the development of mood and other disorders. In support of this perspective, baseline SII, an objective marker of the balance between host systemic inflammation and the immune response status was positively associated with measures of anxiety and depression at follow-up in COVID-19 patients (9). Furthermore, plasma CRP levels (marker of systemic inflammation) have been shown to correlate with deficits on the continuous performance test in recovered COVID-19 patients, suggesting that inflammation may play a role in cognitive complaints in remitted patients (70). Further studies also support the role of inflammation in the development of post COVID-19 syndrome. A study of 121 mild COVID-19 cases found that extended symptomology was associated with higher inflammatory markers [elevated neutrophil, NLR, fibrinogen, and CRP levels (95)]. A study by Yang et al. examined the transcriptomes of eight deceased COVID-19 patients compared to fourteen controls. Despite no molecular evidence of COVID-19 in the brain, they found greater disruptions in all types of cells examined (neurons, glia, immune cells) compared to those that died of alternate causes. Additionally, the sequalae of the COVID-19 patients' brains included inflammation, neurodegeneration, and disrupted signaling, similar to those of other brain-based disorders including schizophrenia, depression, and disrupted cognition (96). These suggest the underlying cellular mechanisms for acute and chronic symptomology such as headaches, mood disorders, brain fog, and cognitive disruptions may be a disruption or dysregulation of neuro-cellular function. Although the degree of neuroinvasiveness and neurological effects of SARS-CoV-2 is still under investigation, it is clear that it generates significant immunological responses in the CNS, which may relate to the development of neuropsychiatric symptomology Figure 1.

## Limitations

There are limitations of this review that need to be acknowledged. Firstly, this is not a systematic review but rather a narrative review, which makes it subject to selection bias regarding the articles included while searching in databases without a structured format. The articles included in the review may also have a few limitations given that research on this topic is currently in nascent stages. As of October of 2021, the World Health Organization established a definition of post-COVID-19 condition (long COVID) through Delphi consensus and requires symptoms not explainable by other means 3 months after infection and persisting for 2 months (1). Many of the papers in our review characterize chronic COVID-19 symptomology differently as the consensus was not yet established at the time of publication. As such, papers featuring chronic symptomology are noted based on the characterizations of the paper's authors and references to “recovery” within the paper. Further, most studies are cross-sectional and hence causality cannot be established. In the same vein, pre-pandemic measures of mood and cognition are not available in most of the studies making it difficult to disentangle the effects of SARS-CoV-2 infection on these measures from pre-existing problems with mood and cognitive ability. In addition, limitations related to measurements and methodological approaches should be considered when interpreting the results. Most of the studies used online surveys and self-reports to assess mood and cognitive ability, which may have limited sensitivity and specificity when compared to structured clinical interviews (97). Finally, it is difficult to disentangle the effects of COVID-19 infection vs. pandemic-related stress since both may contribute to long-term sequelae via neuroimmune mechanisms.

## Future directions

It is reported that pandemic-related sociodemographic factors including isolation, economic uncertainty, and hospitalization have an impact on mental health. Further, it is increasingly evident that the physiological effects of SARS-CoV-2 on the brain and in the periphery of the body have potentially deleterious effects impacting neuropsychological health. Moving forward there is a need for reporting of methods used in determination of COVID-19 populations and increased longitudinal research in recovered COVID-19 patients to investigate the effects of the virus on psychiatric and cognitive health. Prospective studies should utilize comprehensive cognitive and neuropsychiatric assessments along with brain imaging to better understand the long-term CNS effects of COVID-19. Second, a better characterization and understanding of the term “brain-fog” that many “long COVID-19” patients report is needed as the symptoms of the fog relate to many mood symptoms and cognitive deficits that could be detrimental in the long-term. It is necessary to identify any long-term sequelae and provide mental health support and cognitive rehabilitation to minimize the potential negative effects on psychosocial functioning and quality of life of recovered COVID-19 patients. Third, given the increased risk of negative health outcomes in older individuals, future studies should examine and determine whether COVID-19 may trigger or aggravate neurodegenerative processes in this vulnerable group, as an early diagnosis and intervention are the most important strategies to slow the progression of neurodegenerative disorders. One promising area is in the delineation of changes in neuroinflammatory markers to ascertain the role of neuroinflammation in driving the neuropsychiatric symptoms in active and recovered COVID-19 patients (98).

Finally, mechanistic studies that better inform our understanding of the neurobiological features underlying the neuropsychiatric sequelae of COVID-19 infection will help focus the development of therapeutic targets that may help reduce the long-term burden of COVID-19 in recovered patients.

## Conclusions

To date there has been little attention to what may delineate the chronic and acute symptomologies of COVID-19. This review offers a novel investigation into how the neurological effects of SARS-CoV-2 may mediate some of the differences between chronic and acute symptomology. This review suggests that COVID-19 likely has important neuropsychiatric effects in in both the short and longer term. Patients in the acute phase of the illness commonly showed impairment in mood, mania, PTSD-like symptoms, sleep disturbances, and delirium. These symptoms were also prevalent in recovered COVID-19 patients. While sociodemographic and environmental factors such as pandemic-related stress and social isolation undoubtedly play a role in the development of these COVID-19 associated neuropsychiatric syndromes, a number of hypotheses have been proposed highlighting potential neurobiological mechanisms as causal factors for the observed neuropsychiatric symptoms, with inflammatory pathways being the most prominent. The scale of the pandemic will require that brain health be an integral focus of research and clinical service planning in the future.

## Author contributions

CJS developed the concept for the review, conducted the literature search, and drafted the manuscript. CS supported the literature search and supplemented and edited the manuscript. DY-T provided oversight for the manuscript and collaborated on the conceptualization of the project and interpretation of the findings. PR supported and advised on the conceptualization of the project and literature search. All authors reviewed and edited the manuscript.

## Funding

This manuscript is supported by funding from the Utah Science Technology and Research Initiative (USTAR) to DY-T and is also supported by the Medical Research Service of the Veteran Affairs Salt Lake City Health Care System, the Department of Veteran Affairs Rocky Mountain Network Mental Illness Research, Education, and Clinical Center (MIRECC). The views in this paper are those of the authors and do not necessarily represent the official policy or position of the Department of Veteran Affairs or the United States Government.

## Conflict of interest

The authors declare that the research was conducted in the absence of any commercial or financial relationships that could be construed as a potential conflict of interest.

## Publisher's note

All claims expressed in this article are solely those of the authors and do not necessarily represent those of their affiliated organizations, or those of the publisher, the editors and the reviewers. Any product that may be evaluated in this article, or claim that may be made by its manufacturer, is not guaranteed or endorsed by the publisher.
